# Dorsolateral Nigral Hyperintensity on 1.5 T Versus 3 T Susceptibility‐Weighted Magnetic Resonance Imaging in Neurodegenerative Parkinsonism

**DOI:** 10.1002/mdc3.13736

**Published:** 2023-04-14

**Authors:** Anna Grossauer, Christoph Müller, Anna Hussl, Florian Krismer, Michael Schocke, Elke Gizewski, Philipp Mahlknecht, Christoph Scherfler, Gregor K. Wenning, Werner Poewe, Klaus Seppi, Beatrice Heim

**Affiliations:** ^1^ Department of Neurology Medical University of Innsbruck Innsbruck Austria; ^2^ Department of Radiology Medical University of Innsbruck Innsbruck Austria; ^3^ Neuroimaging Research Core Facility Medical University of Innsbruck Innsbruck Austria

**Keywords:** dorsolateral nigral hyperintensity, Parkinson's disease, multiple system atrophy, progressive supranuclear palsy, susceptibility‐weighted imaging

## Abstract

**Background:**

An absent dorsolateral nigral hyperintensity (DNH) is a common finding in patients with neurodegenerative parkinsonism at high or ultra‐high field susceptibility‐weighted magnetic resonance imaging (SWI).

**Objective:**

Despite increasing use of high field magnetic resonance imaging (MRI) in specialized centers, these scanners are still frequently unavailable in primary care or outpatient facilities and underdeveloped or emerging countries. Therefore, the aim of the present study was to evaluate the diagnostic utility of DNH assessment at 1.5 versus 3 T MRI to distinguish patients with neurodegenerative parkinsonism, including Parkinson's disease (PD), multiple system atrophy (MSA) and progressive supranuclear palsy (PSP), from healthy controls (HC).

**Methods:**

Absence of DNH was assessed on visual inspection of anonymized 1.5 T and 3.0 T SWI scans in a case–control study including 86 patients with neurodegenerative parkinsonism and 33 healthy controls (HC). All study participants were consecutively recruited to undergo 1.5 and 3 T MRI.

**Results:**

Overall correct classification was 81.7% (95% CI, 72.6–88.4%) for 1.5 T and 95.7% (95% CI, 89.1–98.7%) for 3 T MRI in discriminating neurodegenerative parkinsonism from controls. However, while DNH was bilaterally present in all but one of the HC at 3 T MRI, it was rated as abnormal (at least unilateral absence) in 15 of 22 HC at 1.5 T MRI, resulting in a specificity of 31.8%.

**Conclusions:**

The results of the present study demonstrate an insufficient specificity of visual assessment of DNH at 1.5 T MRI for the diagnosis of neurodegenerative parkinsonism.

Despite recent refinements in clinical diagnostic criteria, the differential diagnosis of Parkinson's disease (PD) and atypical Parkinsonian disorders (APD), including multiple system atrophy (MSA) and progressive supranuclear palsy (PSP), remains challenging.^1^ Therefore, validated tools and biomarkers are needed to increase the diagnostic accuracy and also to identify PD and APD patients at early or prodromal stages.

Traditionally, magnetic resonance imaging (MRI) has proven to be a valuable diagnostic tool in the differential diagnosis of parkinsonian syndromes as it is capable of detecting causes for secondary parkinsonism and advanced MRI techniques can even deliver signal changes in the basal ganglia and infratentorial structures suggestive for APDs.^2^

A novel MRI marker in the dorsolateral aspect of the substantia nigra pars compacta (SNpc), has been recently investigated as a tool to distinguish PD patients from healthy controls on high or ultra‐high field (3 Tesla or above), iron‐sensitive MRI sequences, including susceptibility‐weighted imaging (SWI).^3^, ^4^, ^5^ Initial reports demonstrated a consistent hyperintense, ovoid signal in the dorsolateral border of the otherwise hypointense pars compacta of the substantia nigra in healthy individuals, resembling the appearance of a swallow‐tail, whereas in patients with neurodegenerative parkinsonism this dorsolateral nigral hyperintensity (DNH) was observed to disappear at least unilaterally on iron‐sensitive MRI sequences.^3^, ^4^, ^5^

Based on the results of a postmortem 7.0 Tesla (T) MRI study, DNH of the SNpc has been postulated to correspond to the area of nigrosome 1 (N1).^4^ The histological concept of nigrosomes was first described by Damier et al.^6^ to refer to calbindin‐poor zones in immunohistochemical staining of the SN. Among a total of five nigrosomes, nigrosome 1 was found to be the area first and mostly affected by neuronal dopaminergic cell loss in PD.^6^, ^7^ Therefore, the loss of DNH in PD, also referred to as N1‐sign,^4^ may have potential as an imaging biomarker to identify patients with neurodegenerative parkinsonism at early or even prodromal stages.

So far, the concept of DNH as an imaging biomarker in neurodegenerative parkinsonism has only been studied more extensively at 3.0 T MRI or scanners with even higher field strengths, with limited evidence for its use at 1.5 T MRI.^8^ Despite increasing use of high field MRI in specialized centers, these scanners are frequently unavailable in primary care or outpatient facilities and underdeveloped or emerging countries. Thus, we aimed to evaluate the diagnostic performance of absent dorsolateral nigral hyperintensity in patients with neurodegenerative parkinsonism, including PD, MSA and PSP, and healthy controls (HC) at 1.5 and 3 T MRI.

## Methods

### Subjects

We consecutively recruited 41 patients with PD, 22 patients with MSA, 23 patients with PSP, and 33 HC to undergo both 1.5 T (Magnetom Avanto; Siemens, Erlangen, Germany) and 3 T MRI (Magnetom Verio; Siemens, Erlangen, Germany). Clinical diagnoses of PD, MSA or PSP were based on established criteria^9^, ^10^, ^11^ and made by movement disorder specialists experienced in parkinsonian disorders. Since all assessments of the study were performed before 2017, PSP patients were classified according to the National Institute of Neurological Disorders and Stroke and Society for PSP (NINDS‐SPSP) criteria. Criteria for PSP were modified by not applying the “first year of disease” specification so as not to exclude PSP‐P (parkinsonian variant of PSP) patients, who were diagnosed according to criteria published in 2010,^12^ as described in our previous study.^5^ All MSA patients presented with parkinsonian features, 21 of the 22 patients with MSA, and 12 of the PSP patients fulfilled the criteria of possible or probable MSA and PSP at the time of MRI scan, whereas nine of the PSP patients were diagnosed as PSP‐P. All patients were clinically followed for at least 24 months. Three patients who were diagnosed as having PD at the time of scanning were reclassified as MSA (1 case) or PSP (2 cases) during clinical follow‐up. Disease severity was assessed using the Hoehn & Yahr (H&Y) staging. Severity of motor symptoms was assessed using the Unified Parkinson's Disease Rating Scale Part III (UPDRS Part III). All patients were examined on regular medication. The data of 3.0 T MRI were already reported previously^5^ from all but one (this was a PSP‐P patient) study participant.

The study was approved by the local Ethics Committee. Written informed consent was obtained from all participants before being enrolled in this study.

### Magnetic Resonance Imaging Protocol

The MRI protocol at 3 T included axial SWI 3D sequences with a TR of 28 ms, a TE of 20 ms, a flip angle of 15, a slice thickness of 2.4 mm and a field of view of 178 × 220 mm. The corresponding voxel size was 0.7 × 0.7 × 2.4 at 3 T MRI. At 1.5 T the MRI protocol included axial SWI 3D sequences with a TR of 49 ms, a TE of 40 ms, a flip angle of 15, a slice thickness of 2.4 mm and a field of view of 192 mm × 220 mm. The corresponding voxel size was 0.9 × 0.9 × 2.4 at 1.5 T MRI. Post processing of the acquired data, including filtering of the phase images, was performed automatically according to an established algorithm by the scanner software after the acquisition was finished. The sequence thus automatically delivered SWI‐filtered phase data as well as SWI magnitude data.

### Imaging Analysis

Symptomatic parkinsonism was excluded by an experienced neuroradiologist (MS) with the routine MR sequences. The visual assessment of DNH was performed using a PACS workstation (Impax‐workstation; IMPAX EE R20 IX CP2 1.1.0.1, AGFA health care N.V., Belgium) by an experienced movement disorders expert with 20 years of experience in MR diagnostics of movement disorders (KS), who was blinded to clinical diagnosis.

Unilateral absence of DNH was regarded as abnormal and therefore indicative of neurodegenerative parkinsonism. Scans were then classified into three groups: “abnormal” (lack of DNH at one side irrespective of the other side) versus “normal” (DNH present at both sides) versus “non diagnostic” (poor quality of SN evaluation at both sides or DNH present at one side and poor quality of SN evaluation at the other side).

For the calculation of interrater reliability, we randomly selected 25 3 T and 51 1.5 T scans for the evaluation of the presence or absence of DNH by two senior (KS, CM) and two junior (AG, BH) investigators. Consensus agreement for scans that were scored differently by the two junior or the senior investigators was sought in a final assessment such as interrater reliability between the consensus rating of the senior and junior raters could be calculated.

### Statistical Analysis

IBM® SPSS® Statistics 27.0 (SPSS, Inc., Chicago, IL, USA) and GraphPad Prism5 (GraphPad Software, Inc., La Jolla, CA, USA) were used for statistical analyses, the latter for calculation of the confidence intervals. Group comparisons for demographic and clinical data were performed using parametric, nonparametric or chi‐square tests depending on the distribution and the scale type of the variables. If normality assumptions were met according to Shapiro–Wilk tests, parametric tests were applied. Otherwise, nonparametric tests were used. The Bonferroni correction was used for all post‐hoc pairwise comparisons.

For analysis of the diagnostic accuracy of an absent DNH in neurodegenerative parkinsonism only scans with a sufficient quality for visual assessment were included. Measures of diagnostic accuracy included sensitivity, specificity, positive predictive values, negative predictive values, likelihood ratios and overall correct classification. Group comparisons for the presence or absence of DNH were carried out using the chi‐square test. If there were cells with a count <5 in the crosstabulation the Fisher's exact test was used instead of Pearson's chi‐square. The 95% confidence interval for the diagnostic accuracy measures was assessed according to the modified Wald method.

The interrater reliability among junior and senior raters were tested with Cohen's kappa statistics. Interpretation of *k* values was based on recommendations by Landis and Koch^13^: 0 to 0.20 slight agreement; above 0.20 up to 0.40 fair agreement; above 0.40 up to 0.60 moderate agreement; above 0.60 up to 0.80 substantial agreement; above 0.80 up to 1.00 excellent agreement.

A two‐sided *p*‐value <0.05 was considered statistically significant.

## Results

### Demographic and Clinical Data

Patient demographics and clinical characteristics are summarized in Table 1. As outlined in Table 1, group differences were observed for age at MRI scan, disease duration at MRI, UPDRS Part III scores and the H&Y stage. HC were significantly younger than patients with PSP. Disease duration was significantly longer in the PD cohort compared to the MSA and PSP cohort, whereas MSA and PSP patients presented with a significantly higher H&Y stage. Additionally, the MSA cohort showed a significantly higher score in the UPDRS Part III rating scale compared to PD patients.

**TABLE 1:** Demographic and clinical data of the study cohort

Group	Sex distribution (female/male)^a^	Age at MRI (years; mean ± SD (median))^b^	Disease duration at MRI (years; mean ± SD (median))^b^	UPDRS Part III (mean ± SD (median))^b^	Hoehn & Yahr Scale (mean ± SD (median))^b^
PD N = 41	15/26	65. 50 ± 9.33 (67.0)	6.58 ± 4.55 (5.5)	27.59 ± 11.59 (26.0)	2.38 ± 0.62 (2.0)
MSA N = 22	9/13	63.24 ± 9.11 (64.3)	1.69 ± 1.36 (1.2)	42.86 ± 9.37 (42.5)	3.39 ± 0.53 (3.0)
PSP N = 23	7/16	68.20 ± 5.78 (68.9)	1.67 ± 1.13 (1.2)	32.00 ± 9.91 (34.0)	3.13 ± 0.73 (3.0)
HC N = 33	20/13	60.46 ± 9.79 (63.1)	NA	1.55 ± 1.99 (1.0)	NA
*p* value for group comparisons	0.094	0.016	<0.001	<0.001	<0.001
*P* values for post‐hoc comparisons					
HC vs. PD	NS	NS	NA	<0.001	NA
HC vs. MSA	NS	NS	NA	<0.001	NA
HC vs. PSP	NS	0.017	NA	<0.001	NA
PD vs. MSA	NS	NS	0.011	0.002	0.002
PD vs. PSP	NS	NS	0.010	NS	0.043
MSA vs. PSP	NS	NS	NS	NS	NS

Abbreviations: MRI, magnetic resonance imaging; SD, standard deviation; UPDRS, Unified Parkinson's Disease Rating Scale; NA, not applicable; NS, not significant.

^a^ Chi‐square test.

^b^ Nonparametric tests (Kruskal‐Wallis 1‐way ANOVA with post‐hoc Mann–Whitney U tests, *p* values were adjusted for multiple comparisons with the Bonferroni correction).

### Dorsolateral Nigral Hyperintensity at 1.5 and 3 T SWI Sequences

Overall, SWI sequences at 1.5 and 3 T MRI scans were analyzable in 93 of 119 study participants (78.1%; PD = 33, MSA = 18, PSP = 20, HC = 22). Poor quality was seen for the evaluation of the absence of DNH in 26 (21.8%) of the study participants at 1.5 T MRI, from which 14 concurrently showed poor quality at 3.0 T MRI (11.8% of the study cohort).

**FIG. 1:**
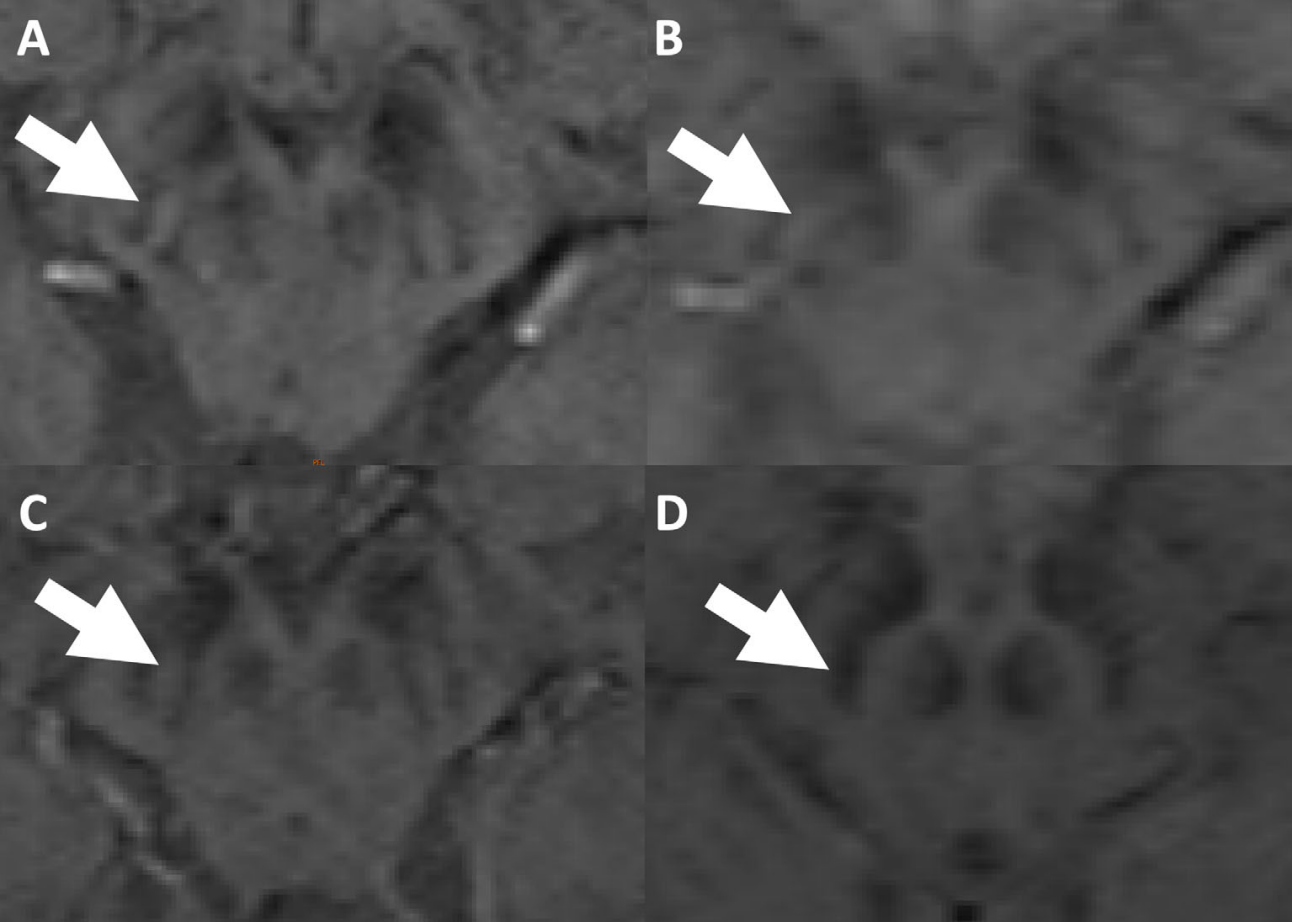
**AB:** SWI images of a healthy person demonstrating DNH at 3 T (**A**) and 1.5 T (**B**). **CD:** SWI images of another healthy person demonstrating DNH at 3 T (**C**), where no DNH is visible at 1.5 T (**D**).

DNH was rated as absent at least unilaterally in all MSA and PSP patients at 1.5 and 3 T MRI, in 31 of 33 (93.9%) PD patients at 1.5 T MRI and in 30 of 33 (90.9%) PD patients at 3 T MRI, resulting in a sensitivity of 97.2% and 95.8%, respectively, for the diagnosis of neurodegenerative parkinsonism. While DNH was rated as bilaterally present in 21 of 22 HC at 3 T MRI, resulting in a specificity of 95.5%, the number of HC identified with a bilaterally normal DNH was only 7 of 22 participants at 1.5 T MRI, resulting in a specificity of 31.8%. Figure 1 shows SWI sequences for the visual assessment of DNH of two HC at 1.5 and 3 T MRI.

The detailed numbers of scans in each group rated with an abnormal DNH at 1.5 and 3 T MRI are given in Table 2.

**TABLE 2:** Diagnostic accuracy of absent DNH for the differentiation of patients with PD, MSA or PSP from HC using 3.0 T MRI

At least unilateral absence of DNH				
PD	MSA	PSP	All PS	HC
30/33	18/18	20/20	68/71	**1** ^**b**^ **/22**

Diagnostic accuracy of at least unilateral absence of DNH				
	PD vs. HC	MSA vs. HC	PSP vs. HC	All PS vs. HC
*p*‐value^a^	< 0.001	< 0.001	< 0.001	< 0.001
Sensitivity	90.9 (75.7–98.1)	100.0 (81.5–100.0)	100.0 (83.2–100.0)	95.8 (88.1–99.1)
Specificity	95.5 (77.2–99.9)	95.5 (77.2–99.9)	95.5 (77.2–99.9)	95.5 (77.2–99.9)
PPV	96.8 (83.3–99.9)	94.7 (74.0–99.9)	95.2 (76.2–99.9)	98.6 (92.2–100.0)
NPV	87.5 (67.6–97.3)	100.0 (83.9–100.0)	100.0 (83.9–100.0)	87.5 (67.6–97.3)
Likelihood Ratio	20.0	22.0	22.0	21.07
Overall correct classification	92.7 (82.3–97.6)	97.5 (86.0–100.0)	97.6 (86.6–100.0)	95.7 (89.1–98.7)

Abbreviations: NPV, Negative predictive value; PPV, Positive predictive value; PS, Parkinsonian syndromes.

*Note*: All measures of diagnostic accuracy (sensitivity, specificity, PPV, NPV, overall correct classification) are given in percent with 95% confidence intervals (CI). CIs were calculated by the modified Wald method.

^a^ Chi‐square or Fisher's exact test.

^b^ The study participant was part of a longitudinal observational study and did neither report nor show any signs and symptoms of degenerative parkinsonism after a follow up of 15 months.

Analyses of all measures of diagnostic accuracy (sensitivity, specificity, positive predictive value, negative predictive value, overall correct classification) for 1.5 and 3 T MRI are summarized in Table 3.

**TABLE 3:** Diagnostic accuracy of absent DNH for the differentiation of patients with PD, MSA or PSP from HC using 1.5 T MRI

At least unilateral absence of DNH				
PD	MSA	PSP	All PS	HC
31/33	18/18	20/20	69/71	**15/22**

Diagnostic accuracy of at least unilateral absence of DNH				
	PD vs. HC	MSA vs. HC	PSP vs. HC	All PS vs. HC
*P*‐value^a^	0.022	0.011	0.009	< 0.001
Sensitivity	93.9 (79.8–99.3)	100.0 (81.5–100.0)	100.0 (83.2–100.0)	97.2 (90.2–99.7)
Specificity	31.8 (13.9–54.9)	31.8 (13.9–54.9)	31.8 (13.9–54.9)	31.8 (13.9–54.9)
PPV	67.4 (52.0–80.5)	54.6 (36.4–71.9)	57.1 (39.4–73.7)	82.1 (72.3–89.7)
NPV	77.8 (40.0–97.2)	100.0 (59.0–100.0)	100.0 (59.0–100.0)	77.8 (40.0–97.2)
Likelihood Ratio	1.378	1.467	1.467	1.425
Overall correct classification	69.1 (55.9–79.8)	62.5 (47.0–75.8)	64.3 (49.1–77.1)	81.7 (72.6–88.4)

Abbreviations: NPV, Negative predictive value; PPV, Positive predictive value; PS, Parkinsonian syndromes.

*Note*: All measures of diagnostic accuracy (sensitivity, specificity, PPV, NPV, overall correct classification) are given in percent with 95% confidence intervals (CI). CIs were calculated by the modified Wald method.

^a^ Chi‐square or Fisher's exact test.

### Interrater Reliability

At 1.5 T, the interrater reliability agreement for the evaluation of DNH between the junior and senior raters was moderate (kappa = 0.42, *p* < 0.001) and substantial (0.74, *p* < 0.001) respectively. On the other hand, at 3.0 T, the interrater reliability agreement for the evaluation of DNH was excellent between both junior (0.81, *p* < 0.001) and senior raters (0.96, *p* < 0.001). Interrater reliability statistics between the consensus rating of the junior versus senior raters revealed moderate agreement for 1.5 T (0.54, *p* < 0.001) and excellent agreement for 3.0 T (0.92, *p* < 0.001).

## Discussion

In the present study, our results demonstrated an insufficient diagnostic accuracy of absent DNH to discriminate patients with neurodegenerative parkinsonism from HC at 1.5 T MRI with a specificity of only 31.8% compared to a specificity of 95.5% at 3 T MRI.

Our study is one of the first to evaluate the diagnostic accuracy of absent DNH among a cohort of patients with PD, MSA or PSP at 1.5 T SWI sequences. To the best of our knowledge, there are only two previous small studies investigating diagnostic accuracy of DNH in study cohorts, where study participants received either 1.5 T or 3.0 T MRI.^8^, ^14^ In one of these studies patients with dementia with Lewy bodies (DLB) were enrolled.^14^ Contrary to these two previous studies,^8^, ^14^ all patients included in the present cohort received 3 T as well as 1.5 T MRI, thus making results for different field strengths directly comparable. While DNH was present in all HC at 3 T MRI in the study by Oustwani et al.,^8^ it was rated as absent in one third of HC at 1.5 T MRI, supporting our results for an insufficient specificity of this imaging marker at 1.5 T MRI. Still, sample sizes in this previous investigation^8^ were extremely small with only 9 controls, 7 PD, 6 MSA and 4 PSP/CBD patients, who were scanned at 1.5 T MRI. In the diagnosis of DLB, 1.5 T MRI also yielded a lower diagnostic accuracy of absent DNH compared to 3 T MRI,^14^ further encouraging the use of higher field strengths for the visual assessment of this imaging marker. Scan parameters for SWI sequences at 1.5 T in these two previous studies^8^, ^14^ were very similar compared to our study.

In accordance with our previous study^5^ interrater reliability of the visual assessment of DNH at 3.0 T was excellent for experienced raters in a newly performed analysis. Here, we were able to show that this is also true for junior raters. On the other hand, at 1.5 T interrater reliability of the visual assessment of DNH was only substantial for senior raters and even only moderate for junior raters. Also considering the low specificity of the visual assessment of DNH at 1.5 T obtained in the present study, we cannot recommend the use of low field strength MRI in the assessment of DNH to differentiate patients with neurodegenerative parkinsonism from healthy controls using routine SWI sequences. Of note, one previous study^8^ demonstrated a higher interrater agreement at 1.5 T than we observed in the present study. However, patient numbers in this previous study^8^ were small. As the visual assessment of DNH should serve as a simple imaging tool in clinical routine to support the diagnosis of neurodegenerative parkinsonism, it is of importance that rating of this sign also achieves good interrater agreement among neurologists with little experience, which could not be achieved at 1.5 T in this study.

The findings of the present study, suggesting an insufficient interrater reliability and specificity of the visual assessment of DNH at 1.5 T MRI, are in line with increasing evidence pointing towards the importance of improved technical standards when evaluating this imaging marker. Notably, more advanced techniques like susceptibility‐map weighted imaging (SMWI), which can be generated from the SWI protocol by combining a magnitude image with a quantitative susceptibility mapping (QSM)‐based weighting factor,^15^, ^16^ were investigated to enhance the visibility of DNH by improving the contrast‐to‐noise ratio (CNR).^16^ A direct comparison of SMWI to standard SWI in the assessment of DNH resulted in a superiority of SMWI in terms of a higher interobserver agreement, a higher concordance rate with ^123^I‐FP‐CIT SPECT and a higher diagnostic performance for PD.^17^ Therefore, our results and the findings of previous studies using more advanced SWI techniques^16^, ^17^, ^18^, ^19^ emphasize the importance of appropriate technical standards to ensure a sufficient diagnostic accuracy of DNH in neurodegenerative parkinsonism. Particularly important in imaging the SN are a sufficient spatial resolution, the slice thickness and the signal‐to‐noise‐ratio (SNR).^20^ Considering these aspects, 7 T MRI composes the best technical standards for imaging of the SN as it provides an increased spatial resolution compared to 1.5 T and 3 T MRI. Additionally, 7 T MRI is more sensitive to magnetic susceptibility effects in iron‐loaded regions than MRI with lower field strengths, resulting in an improved contrast.^21^ Nevertheless, no overt differences in the assessment of DNH with 3 T MRI compared to 7 T devices have been reported.^22^

While an absent DNH is not able to distinguish between PD and atypical parkinsonism, there is limited evidence that imaging of DNH at 3 T MRI can assist in the differential diagnosis of PD and essential tremor^23^ or drug‐induced parkinsonism,^24^ as in the latter two the SN should not be affected by dopaminergic‐neuronal cell loss. Therefore, visual assessment of DNH at high or ultra‐high field strength MRI may be an innovative method in clinical routine to distinguish between neurodegenerative and non‐neurodegenerative parkinsonism and to refine the selection of patients who should receive ^123^I‐FP‐CIT SPECT imaging. This assumption is encouraged by reports of concordance rates about 80% between assessment of DNH using SWI and ^123^I‐FP‐CIT SPECT.^25^, ^26^

Intriguingly, the diagnostic performance of DNH has also already been tested in patients with idiopathic rapid‐eye movement sleep behavior disorder (iRBD), a condition which is well known for its’ risk to convert into an alpha‐synuclein‐related form of neurodegenerative parkinsonism.^27^ Several years ago, we were able to demonstrate an absent DNH in two thirds of patients with iRBD, suggesting the use of this imaging marker for the identification of prodromal degenerative parkinsonism in iRBD.^28^ Two further studies assessing DNH in patients with iRBD investigated an absence of this marker in 27.5%^29^ and 61% of patients,^30^ respectively. Of note, an absent DNH in this patient group seems to correlate with reduced ^123^I‐FP‐CIT uptake,^29^, ^30^ which yields very interesting insights for the development of risk scores to identify PD patients at prodromal stages.

The present study has some limitations: First, we had 26 scans of poor quality mostly due to motion artifacts, in which SWI sequences, which are particularly prone to such artifacts,^31^ were not analyzable to reliably assess DNH on one or both sides. We therefore recommend performing SWI scans at the beginning of the MRI protocol when the assessment of DNH is of interest, as the risk of movement artifacts increases with longer durations spent in the scanner. Secondly, rating of an MRI marker always has a subjective component. However, interrater agreement at 3.0 T MRI was excellent between both junior and senior raters, therefore promoting a reliable use of this imaging biomarker at scanners with high field strength, even when raters have little experience.

Third, due to the absence of postmortem verification misdiagnosis cannot be excluded in some of the clinically diagnosed patients, especially when the disease is at an early stage. However, all patients were followed up for at least two years and a change in diagnosis was only reported in a minority of subjects.

## Conclusion

The results of the current study demonstrate an insufficient specificity of visual assessment of DNH at 1.5 T MRI. While visual assessment of DNH using high field routine SWI sequences serves as a simple diagnostic imaging marker for neurodegenerative parkinsonism, its use at 1.5 T cannot be recommended.

## Author Roles

(1) Research project: A. Conception, B. Organization, C. Execution; (2) Statistical analysis: A. Design, B. Execution, C. Review and Critique; (3) Manuscript preparation: A. Writing of the first draft, B. Review and Critique.

A.G.: 1B, 1C, 2A, 2B, 3A

C.M.: 1B, 1C, 2A, 2B, 3B

A.H.: 1B, 3B

F.K.: 3B

M.S.: 1C, 3B

E.G.: 3B

P.M.: 3B

C.S.: 3B

G.W.: 3B

W.P.: 1A, 1B, 3B

K.S.: 1A, 1B, 1C, 2A, 2B, 2C, 3B

B.H.: 1A, 1B, 1C, 2C, 3B.

## Disclosures

**Ethical compliance statement:** We confirm that we have read the Journal's position on issues involved in ethical publication and affirm that this work is consistent with those guidelines. The study was approved by the ethics committee of the Medical University of Innsbruck. Written informed consent was obtained from all participants before being enrolled in this study.

**Funding Sources and Conflicts of Interest:** This study is supported by funds of the Oesterreichische Nationalbank (Austrian Central Bank, Anniversary Fund; project no.: 14174) and the Austrian Science Fund (FWF: Der Wissenschaftsfonds; project no.: KLI82‐B00). The authors declare that there are no conflicts of interest relevant to this work.

**Financial Disclosures for the Previous 12 Months:** Anna Hussl, Michael Schocke, Elke Gizewski, Philipp Mahlknecht and Christoph Scherfler declare that there are no additional disclosures to report. Anna Grossauer received travel grants from Boston Scientific and Novartis. Christoph Müller received honoraria from Ipsen, Abbvie and Merz. Florian Krismer received personal fees from Institut de Recherches Internationales Servier, Clarion Healthcare, Takeda Pharmaceuticals, Sanofi‐Aventis and the Austrian Society of Neurology; grant support from the National Institutes of Health outside of the submitted work. Gregor K. Wenning received honoraria from Ono, Inhibikase and Takeda. Werner Poewe reports personal fees from: AC Immune, Alterity, AbbVie, BIAL, Britannia, Lundbeck, Neuroderm, Roche, Sunovion, Stada, Takeda, UCB and Zambon (consultancy and lecture fees in relation to clinical drug development programs for PD). Klaus Seppi reports personal fees from Ono Pharma UK Ltd, Teva, UCB, Lundbeck, AOP Orphan Pharmaceuticals AG, Roche, Grünenthal, Stada, Licher Pharma, Biogen, BIAL, EverPharma and Abbvie, honoraria from the International Parkinson and Movement Disorders Society, research grants from FWF Austrian Science Fund, Michael J. Fox Foundation, and AOP Orphan Pharmaceuticals AG. Beatrice Heim reports honoraria from AOP orphan Pharmaceuticals AG and research grants from FWF Austrian Science Fund, outside the submitted work.
